# High-throughput SNP genotyping in *Cucurbita pepo *for map construction and quantitative trait *loci *mapping

**DOI:** 10.1186/1471-2164-13-80

**Published:** 2012-02-22

**Authors:** Cristina Esteras, Pedro Gómez, Antonio J Monforte, José Blanca, Nelly Vicente-Dólera, Cristina Roig, Fernando Nuez, Belén Picó

**Affiliations:** 1 Institute for the Conservation and Breeding of Agricultural Biodiversity (COMAV-UPV), Universitat Politècnica de València, Camino de Vera s/n, 46022 Valencia, Spain; 2 Instituto de Investigación y Formación Agraria y Pesquera (IFAPA). Área de Mejora y Biotecnología de cultivos. Camino San Nicolás 1, 04745, La Mojonera, Almería, Spain; 3 Instituto de Biología Molecular y Celular de Plantas (IBMCP), Universitat Politècnica de València (UPV)-Consejo Superior de Investigaciones Científicas (CSIC), Ciudad Politécnica de la Innovación (CPI), Ed. 8E, C/Ingeniero Fausto Elio s/n, 46022 Valencia, Spain

## Abstract

**Background:**

*Cucurbita pepo *is a member of the Cucurbitaceae family, the second- most important horticultural family in terms of economic importance after Solanaceae. The "summer squash" types, including Zucchini and Scallop, rank among the highest-valued vegetables worldwide. There are few genomic tools available for this species.

The first *Cucurbita *transcriptome, along with a large collection of Single Nucleotide Polymorphisms (SNP), was recently generated using massive sequencing. A set of 384 SNP was selected to generate an Illumina GoldenGate assay in order to construct the first SNP-based genetic map of *Cucurbita *and map quantitative trait *loci *(QTL).

**Results:**

We herein present the construction of the first SNP-based genetic map of *Cucurbita pepo *using a population derived from the cross of two varieties with contrasting phenotypes, representing the main cultivar groups of the species' two subspecies: Zucchini (subsp. *pepo*) × Scallop (subsp. *ovifera*). The mapping population was genotyped with 384 SNP, a set of selected EST-SNP identified *in silico *after massive sequencing of the transcriptomes of both parents, using the Illumina GoldenGate platform. The global success rate of the assay was higher than 85%. In total, 304 SNP were mapped, along with 11 SSR from a previous map, giving a map density of 5.56 cM/marker. This map was used to infer syntenic relationships between *C. pepo *and cucumber and to successfully map QTL that control plant, flowering and fruit traits that are of benefit to squash breeding. The QTL effects were validated in backcross populations.

**Conclusion:**

Our results show that massive sequencing in different genotypes is an excellent tool for SNP discovery, and that the Illumina GoldenGate platform can be successfully applied to constructing genetic maps and performing QTL analysis in *Cucurbita*. This is the first SNP-based genetic map in the *Cucurbita *genus and is an invaluable new tool for biological research, especially considering that most of these markers are located in the coding regions of genes involved in different physiological processes. The platform will also be useful for future mapping and diversity studies, and will be essential in order to accelerate the process of breeding new and better-adapted squash varieties.

## Background

The *Cucurbita *genus, of American origin, is one of the most variable genera within the Cucurbitaceae family (reviewed by Esteras et al. [1]). *C. pepo *L. (2 n = 40), the most economically important crop of this genus [2], displays eight commercial morphotypes grouped into two subspecies (subsp. *pepo *L.: Pumpkin, Vegetable Marrow, Cocozelle and Zucchini; subsp. *ovifera *(L.) Decker (syn subsp. *texana *(Scheele) Filov): Scallop, Acorn, Crookneck and Straightneck). The main economic value of the species resides in the consumption of its immature fruits as vegetables, commonly known as summer squashes. Summer squashes of the Zucchini type rank among the highest-valued vegetables worldwide, whereas the "winter squash" types (fruits consumed when mature) of *C. pepo *and related *Cucurbita *spp. are food staples and rich sources of fat and vitamins in developing countries [3].

Despite its economic importance, there are few genomic tools available for *Cucurbita*, unlike other cucurbits, such as watermelon (*Citrullus lanatus *(Thunb.) Matsum & Nakai), cucumbers (*Cucumis sativus *L.) and melons (*Cucumis melo *L.), for which new mapping populations, dense genetic maps [4-7], microarrays [8,9], reverse genetics platforms [10,11], transcriptomes [12,13] and even whole genome sequences have already been generated [14-16]. Many of these resources are available at the database maintained by the International Cucurbit Genomics Initiative (ICuGI, [17]) and are being successfully employed by cucurbit researchers to study gene functions and their related polymorphisms.

High-throughput sequencing technologies, mainly Roche 454 and Illumina GA [18], are contributing to filling this gap for non-model crops, thereby allowing the rapid generation of sequence information, even in species about which there is little prior knowledge. One of the most interesting applications of massive sequencing is the large-scale discovery of genetic variants that can be converted into genetic markers, mainly microsatellites or Simple Sequence Repeats (SSR) and Single Nucleotide Polymorphisms (SNP) [19]. SSR and SNP are now the predominant markers in plant genetic analysis. The first transcriptome of *C. pepo *was recently generated using 454 GS FLX Titanium technology. A total of 49,610 unigenes were assembled from 512,751 new EST (Expressed Sequence Tags) and used to generate the first large collection of EST-derived SSR and SNP in this species [20]. SNP are abundant in the genomes, and are stable, amenable to automation and increasingly cost-effective, and are therefore fast becoming the marker system of choice in modern genomics research. SSR, however, continue to be widely used in studies with no need for automation due to their co-dominant and multiallelic nature.

A practical way of optimizing large SNP collections is that of using them with cost-effective platforms for medium- to high-density genotyping. A large number of commercial platforms for SNP genotyping are currently available (reviewed by Gupta et al. [21]). The Illumina GoldenGate assays that genotype 384, 768 or 1,536 SNP in parallel have been the most widely used for mid-throughput applications [22]. This genotyping technique has been used extensively in humans [23] and several animal species [24-26]. SNP platforms are also available for several plant species, made up mostly of cereals, legumes and conifers [27-35]. One of their main applications is the rapid development and saturation of genetic maps [36,37].

Dense genetic maps are necessary tools for efficient molecular breeding. They are particularly useful for quantitative trait *loci *(QTL) mapping and for the development of new high-quality mapping populations, such as introgression line libraries [38,39]. Four genetic maps have been reported in the *Cucurbita *genus to date. The first two maps were constructed using a population derived from an inter-specific cross between *C. pepo *x *C. moschata *Duchesne, a closely related species, with Random Amplified Polymorphic DNA (RAPD) markers [40,41]. Two maps were subsequently produced from two intra-specific crosses, one using a cross between the oil-seed Pumpkin × Zucchini "True French" varieties (both of which belong to *C. pepo *subsp. *pepo*), and the other using a *C. pepo *subsp. *pepo *x *C. pepo *subsp. *ovifera *cross (oil-seed Pumpkin × Italian Crookneck, respectively). These maps consisted mainly of RAPD and Amplified Fragment Length Polymorphisms (AFLP) [42,43]. These markers are dominant and cannot be transferred readily to other populations. The first collection of SSR markers was recently produced from genomic libraries in *Cucurbita *by Gong et al. [44]. Part of this collection, consisting of 178 SSR, was used to increase the density of the Pumpkin × Crookneck map and also to study macrosynteny with *C. moschata *[45]. Before the study by Blanca et al. [20], no SNP were available for the species, which is why these markers have not previously been used for mapping purposes.

Even though nearly one hundred major genes controlling different aspects of *Cucurbita *biology have been described [46], most have not been mapped. The available maps only include a few monogenic traits and have not yet been efficiently used for QTL mapping. There is a growing need for generating new maps with more informative and transferable markers that are amenable to large-scale genotyping. Markers linked to traits of interest are necessary for molecular breeding in these species, mainly in the Zucchini type, which by far dominates the squash market and the breeding efforts of seed companies. The current availability of a collection of 19,980 EST-SNP, located mostly in gene-coding regions [20], will facilitate map development with functional markers.

In this study, we used a set of 9,043 EST-SNP that were detected *in silico *by Blanca et al. [20], and which are suitable for detecting polymorphism between two main commercial types of *C. pepo *(Zucchini and Scallop) that have contrasting vine, flowering and fruit phenotypes, in order to develop an Illumina GoldenGate 384-SNP platform. This platform was employed to build the first SNP-based genetic map with an F_2 _population (Zucchini × Scallop) and to detect QTL for the very first time. The genotyping platform and the genetic map are invaluable new tools for molecular breeding in *Cucurbita*.

## Methods

### Plant material

**Figure 1:**
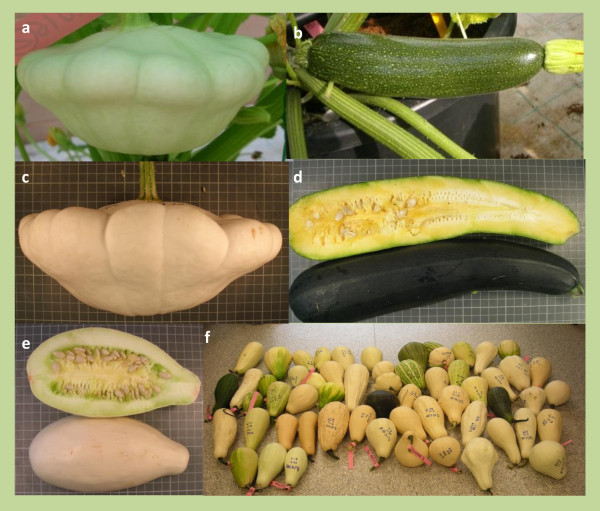
**Fruit characteristics of the map parentals and derived populations**. Pictures showing fruit characteristics of Scallop and Zucchini parentals and derived populations. Immature fruits of Scallop UPV-196 and Zucchini MU-CU-16 (a and b), mature fruits of Scallop UPV-196 and Zucchini MU-CU-16 (c and d), mature fruits of F_1 _(e) and a sample of mature fruits of the F_2 _population (f).

An F_2 _population of 146 plants derived from the *C. pepo *subsp. *pepo *var. Zucchini MU-CU-16 × *C. pepo *subsp. *ovifera *var. Scallop UPV-196 cross was used to generate the linkage map. These are the same parental genotypes that were previously employed to generate the first *C. pepo *transcriptome [20]. Both represent the main summer squash cultivar groups of each subspecies, and have contrasting phenotypes for vine, flowering and fruit traits (Figure 1). Four F_1 _plants and several individuals of each backcross generation to MU-CU-16 (BCZ, 30) and to UPV-196 (BCS, 30) were also included in the assay. In order to check if the selected SNP might also be useful for genetic diversity studies and genotyping in other mapping populations, a panel of seven accessions of *C. pepo*, including representatives of the four morphotypes of the subspecies *pepo *(two Zucchini landraces from southern Spain, MU-20 and E-27; one Vegetable Marrow from Morocco, AFR-12; one Spanish Cocozelle landrace, V112; and two Pumpkin accessions, Styrian Pumpkin and the Mexican landrace, CATIE 18887) and one morphotype of the subspecies *ovifera *(the cultivar Early Summer Crookneck) were included in the genotyping assay. One accession of the related species, *C. moschata*, was also genotyped (the Spanish landrace AN-45). All these accessions belong to the *Cucurbita *core collection of the Cucurbits Breeding Group of the Institute for the Conservation and Breeding of Agricultural Biodiversity (COMAV) [47,48] except for CATIE 18887, which was kindly provided by the Genebank of the Centro Agronómico Tropical de Investigación y Enseñanza (CATIE) in Costa Rica.

Total DNA was extracted from young leaves using the CTAB method [49], with minor modifications. To improve the quality of the obtained DNA, 70% ethanol containing 15 mM ammonium acetate was used in the last wash, and the DNA was treated with RNase. DNA concentrations in TE buffer were adjusted to 50 ng/μl, with the PicoGreen fluorescence being measured on an ABI7900 apparatus (Applied Biosystems). Samples were sent for genotyping to the Centro Nacional de Genotipado (CEGEN-ISCIII, CRG-Node, Barcelona), a high-throughput genotyping service. A minimum of 200 ng of DNA were used for SNP genotyping.

### SNP selection for the GoldenGate platform

Using the first *C. pepo *transcriptome [20] as a reference, a collection of 512,751 *C. pepo *EST, generated using 454 pyrosequencing, from the two genotypes used as the parentals for the mapping population (Zucchini MU-CU-16 and Scallop UPV-196), was mined for SNP. This screening yielded a total of 19,980 putative SNP and 1,174 INDEL, distributed in 8,147 unigenes. Using the different filters established in [20], we selected a set of markers that, *in silico*, were monomorphic within and polymorphic between the two sequenced genotypes and suitable for genotyping with the Illumina GoldenGate system. Only SNP were selected, as the INDEL were discarded [20]. Sequences with more than 4 SNP or INDEL per 100 bp were discarded (using filter HVR4) to avoid SNP located in hypervariable regions. This selection was intended to reduce false polymorphisms caused by the alignment of paralogs, a potentially significant problem when aligning short sequence reads. To facilitate their use in a GoldenGate genotyping assay, we also discarded those SNP that were closer than 60 bp to another SNP or INDEL, to an intron or to the unigene edge (filtering them out with CS60, I60 and CL60, respectively). Only SNP with two or more reads per allele were selected, since our previous experience with *in silico*-detected SNP in melon [13] indicated that putative SNP with only one read in one allele have a low percentage of validation (even when the quality of the sequenced nucleotide is high). Blanca et al. [20] annotated the unigene collection using the Blast2GO package [50], which assigns Gene Ontology (GO) terms based on the BLAST definition. We used this annotation to prioritize the selection of SNP located in the Open Reading Frame regions (ORF) of annotated unigenes (with GO terms and significant BLAST in the Swiss-Prot, *Arabidopsis *org or Uniref90 databases [51-53] and with orthologs of *Arabidopsis *and/or melon). A set of SNP that generate allele-specific restriction targets, with the possibility of being detected *via *Cleaved Amplified Polymorphic Sequences (CAPS), was also included, even though they did not meet some of the aforementioned requirements.

The GO terms were reclassified into different functional groups based on a set of GO slims in the Molecular Function and Biological Process categories in order to provide a broad overview of the ontology content of the final platform.

### SNP genotyping

The sequence of each selected *locus*, including the polymorphic nucleotide and a 60 bp flanking sequence, was submitted to the Illumina Assay Design Tool (ADT) (Illumina, San Diego, CA), and designability scores were used for final marker selection. These scores ranged from 0 to 1.0, where a score of > 0.6 means a high success rate for the conversion of an SNP into a successful GoldenGate assay. On the basis of these scores, a final set of 384 SNP was selected, which was predicted to have a high likelihood of success. The GoldenGate genotyping assay was conducted as described elsewhere [22,28,33].

To summarize, three primers were designed for each *locus*. Two were allele-specific oligos (ASOs), complementary to the sequence directly adjacent to the SNP, only differing at the 3' base complementary to each allele. The third primer was a *locus*-specific oligo (LSO), which hybridizes to the complementary sequence located downstream of the target SNP. The three oligos had three universal primers attached at the 5' end. Each *locus*-specific oligo also had an "IllumiCode" sequence complementary to the array. The sequence of each *locus *and the 1,152 custom oligos, three at each of the 384 different SNP *loci*, are listed in Additional File 1: **"**Sequence and primers for genotyping the 384 SNP included in the GoldenGate platform".

After DNA hybridization, an extension and ligation step was performed connecting each allele-specific oligo with the *locus*-specific oligo. A PCR step was then conducted for all 384 *loci *using common universal primers. The GoldenGate assay was deployed on the BeadXpress^R ^platform using Veracode^R ^technology (Illumina, San Diego, CA) [54]. The PCR products, labeled Cy3 or Cy5 depending on the allele, were hybridized to glass Veracode micro-beads, each bearing a *locus*-specific barcode *via *the corresponding Illumicode sequence. Then, each SNP was identified by its IllumiCode and alleles were discriminated by their fluorescent signals on a Veracode BeadXpress Reader [55].

The automatic allele calling for each *locus *was accomplished using the GenomeStudio software (Illumina, San Diego, CA). The clusters were manually edited when necessary.

### SSR selection and amplification

A set of 25 genomic SSR (gSSR), evenly distributed in the previously published map constructed by Gong et al. [44] using the F_2 _*C. pepo *subsp. *pepo *oil-Pumpkin variety "Lady Godiva" × *C. pepo *subsp. *ovifera *Crookneck variety "Bianco Friulano", were selected to be used as anchors between both maps. Information about the selected SSR is included in Additional File 2: **"**Primers for genotyping the SSR included in the map".

PCR reactions were carried out in a total volume of 15 μl in PCR buffer 1× (75 mM Tris-HCl pH 9, 20 mM (NH_4_)_2_SO_4_, 50 mM KCl), 3 mM MgCl_2_, 200 μM each dNTP, 0.15 μM each primer, 0.2 μM M13 IRDye700/800 (LI-COR. Lincoln, Nebraska) tagged tail, 0.35 U Taq DNA Polymerase (Biotools B&M Labs, S.A., Madrid, Spain) and 10-15 ng DNA. Forward primers were designed with an added M13 tail sequence at their 5' end. The thermal profile was the following for all the *loci*: 3 min denaturation at 95°C, 10 cycles of 30 s at 95°C, 30 s at 65°C (decreasing 1°C every cycle) and 30 s at 72°C, 20 cycles of 30 s at 95°C, 30 s at 55°C and 30 s at 72°C with a final extension of 5 min at 72°C. A LICOR 4300 analyzer was employed to visualize SSR-allele size differences on a denaturing polyacrylamide gel, loading a 1/10 or 1/20 dilution in formamide.

### Linkage analysis and map construction

The genetic map was constructed using the genotyping results for the F_2 _Zucchini × Scallop mapping population, obtained with the new 384-SNP GoldenGate platform and the anchor SSR. Segregation distortion at each marker *locus *was tested against the expected ratio for F_2 _(1:2:1) using a χ^2 ^test. The linkage map was generated with MAPMAKER/EXP version 3.0b [56]. Markers were associated with the "group" command with LOD> 4. Markers within groups were ordered using the "order" command. Distances in centiMorgans (cM) were calculated from the recombination frequencies using the Kosambi mapping function [57]. The remaining markers were then located with the "try" command. The map was drawn with MapChart version 2.1 [58].

### Synteny with cucumber

The colinearity of the *C. pepo *genetic map with the cucumber genome was evaluated by doing a BLAST search of the unigenes corresponding to every *C. pepo *SNP against the cucumber genome. The FASTA sequence of this genome was downloaded from the ICuGI database [17]. The hits obtained in the tBLASTx search of the *C. pepo *unigenes against the cucumber genome were considered to be significant if they had an e-value above 10^-6^. The locations of these significant hits were plotted in a scatter plot in which one axis represented the cucumber genome and the other the *C. pepo *map. The processing of the BLAST results was carried out with a custom Python script that is available upon request.

### Phenotyping

All F_2_, BCZ and BCS plants were cultivated in a greenhouse with a fully randomized experimental design (February to July, 2010) and extensively phenotyped. Five plants of each parental and the F_1 _generation were also included in the assay. Fifty traits were measured for each single plant, and twelve were scored visually (Table 1). Vine traits were related to plant color, length and branching intensity, and flowering traits were related to the flowering time and male/femaleness tendency. Each plant was selfed and two fruits per plant were analyzed. One fruit per plant was analyzed when immature, 7 days after pollination, which corresponds to the commercial state of summer squashes. The second fruit per plant was analyzed at physiological maturity (ranging from 20 to 60 days after pollination). Traits measuring fruit size, shape, texture, firmness, rind and flesh color, sugar content and acidity were analyzed. More details about quantitative and qualitative traits are included in Table 1. Correlations between pairs of traits were estimated by using the Pearson correlation coefficient.

**Table 1:** Quantitative and Qualitative traits measured/scored in the mapping populations

Trait code	Description/categories
**Quantitative traits**	

*Vine traits*	

N°Br	Number of branches 7 days after the appearance of the first female flower

PLe	Plant length measured at the end of the assay (cm)

NoN°	Number of nodes measured at the end of the assay

*Flowering traits*	

NoMaF	First node with a male flower

NoFeF	First node with a female flower

DMaF	Days from sowing to the development of the first male flower

DFeF	Days from sowing to the development of the first female flower

N°MaF	Number of male flowers measured 7 days after the opening of the first female flower

N°FeF	Number of female flowers measured 7 days after the opening of the first female flower

TN°F	Total number of flowers measured 7 days after the opening of the first female flower

MaF/FeF	Ratio male to female flowers

*Immature fruit*	

IPeLe	Peduncle length (mm)

IFLe	Fruit length (cm)

IFWi	Fruit width (cm)

IFWe	Fruit weight (g)

IRTh	Rind thickness (mm)

IFTh	Flesh thickness (mm)

ICaTh	Cavity thickness (mm)

IBrix	Total soluble solids measured with refractometer (Brix degrees)

IRFi	Rind firmness measured with penetrometer (kg)

IFFi	Flesh firmness measured with penetrometer (kg)

IRBr	Rind brightness, scored visually as matte (0), medium (1) and bright (2)

ILoN°	Number of locules

ILRCo	Rind color measured with colorimeter, Hunter parameter L (Lightness: from white, L = 100, to black, L = 0)

IaRCo	Rind color measured with colorimeter, Hunter parameter a (from redness for positive values to greenness for negative values)

IbRCo	Rind color measured with colorimeter, Hunter parameter b (from yellowness for positive values to blueness for negative values)

ILFCo	Flesh color measured with colorimeter, Hunter parameter L

IaFCo	Flesh color measured with colorimeter, Hunter parameter a

IbFCo	Flesh color measured with colorimeter, Hunter parameter b

*Mature fruits*	

DMa	Days from pollination to maturity

MPeLe	Peduncle length (mm)

MFLe	Fruit length (cm)

MFWi	Fruit width (cm)

MFWe	Fruit weight (g)

MRib	Intensity of fruit ribbing, scored visually based on presence and depth of the ribs as absent (0), surface ribbing (1), intermediate ribbing (2) and strong ribbing (3)

MRTh	Rind thickness (mm)

MFTh	Flesh thickness (mm)

MCaTh	Cavity thickness (mm)

MBrix	Total soluble solids measured with refractometer (Brix degrees)

MpH	pH measured with paper

MRFi	Rind firmness measured with penetrometer (kg)

MFFi	Flesh firmness measured with penetrometer (kg)

MRBr	Rind brightness, scored visually as matte (0), medium (1) and bright (2)

MLoN°	Number of locules

MLRCo	Rind color measured with colorimeter, Hunter parameter L

MaRCo	Rind color measured with colorimeter, Hunter parameter a

MbRCo	Rind color measured with colorimeter, Hunter parameter b

MLFCo	Flesh color measured with colorimeter, Hunter parameter L

MaFCo	Flesh color measured with colorimeter, Hunter parameter a

MbFCo	Flesh color measured with colorimeter, Hunter parameter b

**Qualitative traits**	

*Vine traits*	

SC	Stem color, scored as dark green, intermediate or light green

LIns	Green to white color change in leaf insertion scored as absent or present

T	Presence of tendrils scored as absent or present

*Immature fruit*	

IFSh	Fruit shape, scored as elongated, pear-shaped, discoid or oval

IPriRCo	Primary rind color, scored as dark green, green, light green, white-green or white

IPSecRCo	Pattern of secondary color, scored as dotted speckled, striped or absent

IFCo	Flesh color, scored as green, light green, white-green or white

*Mature fruit*	

MFSh	Fruit shape, scored as elongated, pear-shaped, discoid or oval

MPriRCo	Primary rind color, scored as black, green, orange, yellow, cream or white

MPSecRCo	Pattern of secondary color, scored as dotted speckled, banded, striped or absent

MFCo	Flesh color, scored as green, orange, yellow, cream or white

MRTe	Rind texture, scored as smooth or warted

### QTL analysis

QTL for quantitative traits were analyzed by composite interval mapping with Windows QTL Cartographer 2.5 [59] using the developed genetic map and the stepwise forward regression procedure with a walking speed of 1 cM, a window size of 15 cM and the inclusion of up to 5 maximum background marker *loci *as QTL cofactors. The LOD threshold for a Type I error P < 0.05 value was calculated by a permutation test [60] implemented in Windows QTL Cartographer with 1,000 permutations independently for each trait. Additive and dominant QTL effects (a and d, respectively), the degree of dominance (d/[a]) and the proportion of phenotypic variance explained by QTL (R^2^) were estimated at the highest peaks depicted by the QTL analysis.

A positive value of additive effects (positive a) indicates that the Zucchini allele increases the trait, and, conversely, a negative value indicates the Scallop allele increases the trait. For positive a values, positive values of d indicate that the Zucchini allele is dominant, whereas negative values indicate that the Scallop allele is dominant. Conversely, for negative a values, positive and negative d values indicate dominance of the Scallop and Zucchini alleles, respectively.

In order to validate the QTL effects and the utility of the linked markers for breeding purposes (Marker-Assisted Selection, MAS), genotypic and phenotypic data of the two backcross populations, BCZ and BCS, were analyzed for the detected QTL. ANOVA analysis conducted using the SPSS v. 16.0 software was employed to detect significant differences in the average value of homozygous backcross individuals (Zucchini, a, or Scallop, b) *versus *heterozygous individuals (h) for the markers located within or near the 1-LOD interval for the QTL. In those traits displaying QTL confirmed in the backcrosses, broad-sense heritabilities were estimated as described by Wright [61]: H^2 ^= [V_F2 _- (0.25V_Z _+ 0.25V_S _+ 0.5V_F1_)]/V_F2_, where V is the variance of F_2_, Zucchini (Z), Scallop (S) and F_1 _populations, respectively.

For the QTL analysis, the qualitative traits were coded as dummy variables, absent (0) or present (1), and analyzed with the Qgene v. 4.3.9 software [62] using composite interval mapping analysis and conducting 1,000 permutations to calculate the LOD threshold value for P < 0.05 using a resampling test. In order to confirm the observed linkage with the flanking markers, a contingency χ^2 ^test was conducted in those cases in which significant LOD values were found. A null hypothesis (H_0_) of independence of frequency between a trait (scored as 0-1) and the marker (genotyped as homozygous or heterozygous) was checked for the F_2_, with an error type I rate of α = 0.05 and 2 degrees of freedom (df). BCZ and BCS populations were also used for validating QTL effects in qualitative traits. We checked the frequency of the corresponding category in each group of individuals classified according to their genotype for the corresponding linked markers. The association between trait categories and linked markers was also checked using the Fisher exact probability test, as the number of individuals was too low [63,64]. P was calculated as the probability of the observed array of cell frequencies plus the sum of the probabilities of all other cell-frequency arrays that are smaller than the probability of the observed array. H_0 _of independence was rejected when P < 0.05.

Information on those QTL for quantitative and qualitative traits that were validated in the backcrosses was also included in the MapChart file to obtain a more complete map of the species.

## Results and discussion

### Design of the 384-SNP GoldenGate genotyping platform

Of the 19,980 SNP identified *in silico *[20], 9,043 were monomorphic within and polymorphic between the two parents of the map (Zucchini MU-CU-16 and Scallop UPV-196) (Figure 1) and were not located in highly variable regions (filtered out with HVR4). A total of 3,538 of these high-confidence SNP met the criteria for high-throughput genotyping platforms, i.e., being absent of any other known SNP in their vicinity and having enough sequence information up- and downstream of the SNP (filtered out with CS60, CL60 and I60). A preliminary set of 713 SNP, located in different unigenes, was selected, prioritizing SNP in long unigenes with well-defined functions. Designability scores were then given to each *locus *using the Illumina ADT. Only SNP with scores of > 0.6 were selected. Sequences and primers of the finally selected SNP collection are included in Additional File 1. The Illumina scores and annotation details of the corresponding unigenes are also described in Additional File 3: "Annotation data and map position of the 384 *loci *included in the GoldenGate platform".

The final set of the 384 SNP included in the GoldenGate platform had a mean designability score of 0.89. The average length of the selected unigenes was 1,057 bp (ranging from 398 to 2,336). These unigenes were previously annotated [20]. Most SNP (367, 95.6%) were located in the ORF of the corresponding unigene, with only 17 in the untranslated regions (UTR).

Blanca et al. [20] functionally classified the unigenes following the Gene Ontology (GO) scheme. Only 24 of the 384 selected unigenes (6.25%) with SNP could not be assigned to any GO term. We used the GO annotations to assign most unigenes (360, 93.8%) to a set of GO slims in the Biological Process and Molecular Function categories (Additional File 4: "Number of unigenes in each functional category"). The GO annotations for the unigenes showed a fairly consistent sampling of functional classes, indicating that these SNP markers represent genes with different molecular functions and that they are involved in various different biological processes. Cellular, metabolic, biosynthetic and developmental processes were among the most highly represented groups under the Biological Process category (Additional File 4). Other abundant assignments were transcriptional regulation, translation, signal transduction, transport and oxidation-reduction functions. Stimulus, stress- and defense-responsive genes were also well represented. Genes involved in other important biological processes, such as growth, ripening and hormone-signaling processes were included. Some of these genes might play a role in the response to diseases, floral sex determination and fruit development and quality. Under the Molecular Function GO hierarchy (Additional File 4), assignments were mainly to catalytic and binding activities. A large number of hydrolases, kinases and transferases, representing genes involved in the secondary metabolite synthesis pathways, were also included. Transcription and translation factors were also well-represented.

The putative orthologs of all the unigenes were identified [20] by doing a reciprocal BLAST search of the *Arabidopsis *and melon databases [52,17]. Most unigenes selected for the GoldenGate platform had an *Arabidopsis *ortholog (236, 61.4%) and/or a melon ortholog (228, 59.4%). Only 22.4% had no orthologs. GO terms, gene description and a list of the identified orthologs are included in Additional File 3.

### Genotyping results: allele call and polymorphism

The GoldenGate genotyping assay was carried out successfully, with 90.1% of the SNP successfully genotyped taking into account both monomorphic and polymorphic SNP. Only 38 of the 384 SNP included in the platform failed to give a clear genotype. Fifteen and sixteen SNP could not be analyzed due to the absence of or low cluster separation, respectively; five displayed more than three clusters and two had low intensities, according to quality Veracode genotyping (Additional File 3). The absence of cluster separation might be the result of a non-allele-specific match of the primers. Likewise, the existence of more than two alleles and/or the amplification of a non-unique genomic region might be the cause of the existence of more than three clusters.

The average designability score values for failed markers was significantly lower than that of the successful markers (0.86 *versus *0.89, P < 0.05), but all scores were > 0.6, which is considered to be the optimal threshold for a GoldenGate assay. The percentage of failed markers with only 2 reads in one or both alleles (according to the sequencing results, [20]) was higher compared to that of the successful SNP (65.8% *versus *43.6%).

All in all, a total of 346 SNP were classified as successful assays. Similar success rates have been reported in soybean [28], barley [65], maize [37] and pea [33]. All of these markers amplified in nearly all the accessions of *C. pepo*. Knowing their polymorphism in diverse germplasm could help to determine their usefulness in future genetic diversity studies or mapping efforts.

One hundred and ninety-six SNP detected variation among the morphotypes of *C. pepo *subsp. *pepo *(Zucchini, Vegetable Marrow, Cocozelle and Pumpkin), making them useful for genetic diversity studies or for mapping purposes using intra-subspecific crosses. Eighty-two detected variability among the assayed Zucchini types. Zucchini is by far the most important commercial type of summer squash and at the same time the most recently developed and the least variable. Therefore, markers detecting variability within this culti-group could be of interest for cultivar fingerprinting. Fifty-nine SNP detected variation between the two accessions of *C. pepo *subsp. *ovifera *(Crookneck and Scallop) and seventy-eight markers yielded alleles exclusive to only one subspecies. The latter could be of interest for mapping purposes using inter-subspecific crosses. We included two accessions in our assay that belong to the morphotypes of the map parentals of Gong et al. [44] (Styrian Pumpkin and Early Summer Crookneck). Two hundred and fourteen markers were polymorphic between them, and may be used to increase the density of that map, connecting both maps with common markers.

In addition, 305 SNP (79%) were amplified in the *C. moschata *accession. This is an interesting result as most of the platform's markers could potentially be used in introgression programs aimed at transferring traits from *C. moschata *into *C.pepo*. The only previous set of markers that proved to be transferable between *C. pepo *and *C. moschata *was a set of 76 genomic SSR used to perform the first macrosynteny studies between the two species [45]. Our set of functional markers will be useful for further macrosynteny studies with this species and for the marker-assisted selection of traits introgressed from *C*. *moschata *into *C. pepo*.

Details about the polymorphism detected by each SNP are included in Additional File 3. In order to facilitate the future application of this marker set, information about possible detection *via *CAPS is also provided. Sixty-two of the 384 SNP affected restriction targets and could be easily assayed as CAPS.

The 384-SNP set was selected *in silico *for being polymorphic between Zucchini MU-CU-16 and Scallop UPV-196 [20]. Of the 346 successfully called SNP, 330 were polymorphic between these genotypes, yielding 3 clear clusters representing the two homozygous plus the heterozygous genotypes. Sixteen did not show polymorphism between the parentals. This could be explained by artifacts generated during the sequencing process. However, the lack of polymorphism could also be explained by the incapacity of this technique to discriminate an SNP at this *locus*, for example, because of the lack of amplification of one allele due to polymorphism in the priming site. In order to reduce false SNP, we only selected SNP with two or more reads per allele. Most of the monomorphic markers have only 2 reads in one or both alleles (81.3%). These results suggest that a higher number of reads per allele is a good criterion for selecting true SNP from *in silico*-mined collections.

### SSR results

The microsatellite transferability rate from the previous *Cucurbita *map [44] to our mapping population proved to be low, as only 17 out of 25 SSR (68%) amplified, and only 11 (44%) could be mapped. SSR that displayed several amplification problems, such as nonspecific amplification (CMTp235 and CMTp245) and preferential amplification of the MU-CU-16 allele (CMTp86, CMTp188, CMTp47, CMTp256, CMTp33 and CMTp208), were discarded. The preferential amplification of the MU-CU-16 parental is consistent with the origin of these genomic SSR, which were developed from a genomic library derived from a Pumpkin genotype (subsp. *pepo*). Four of the 17 amplified markers were monomorphic in our parentals and two resulted in a distorted F_2 _segregation. Details of the SSR results are included in Additional File 2. SSR are multiallelic markers, easily used by single PCR. However, SSR genotyping cannot be automated and the analysis of large populations is still time-consuming. This makes SNP the preferred markers for different high-throughput genotyping purposes.

### Genetic map of the Zucchini × Scallop population

**Figure 2:**
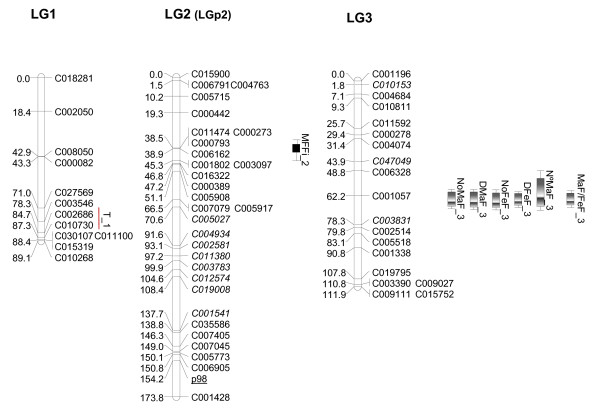
**Genetic map of Zucchini × Scallop F_2 _population (LG1, LG2, LG3)**. Linkage map and locations of quantitative trait *loci *(QTL) whose effects have been verified in the backcross populations associated with vine development, flowering and fruit quality based on 146 F_2 _plants derived from a Zucchini × Scallop cross. The linkage groups (LGs) have been ordered according to the results obtained in this paper. Group numbers in parenthesis (LGp) correspond to LGs in the map by Gong et al. [44]. The correspondence between the two linkage groups has been determined according to the common SSR markers between maps (underlined). Markers with distorted segregation in F_2 _are in italics. QTL indicated in light grey, grey or black correspond to flowering, immature of mature fruit traits, respectively. QTL are represented with bars (2-LOD interval) and boxes (1-LOD interval). QTL for qualitative traits are represented with red lines spanning the region between flanking markers significantly associated with the trait at P < 0.05.

**Figure 3:**
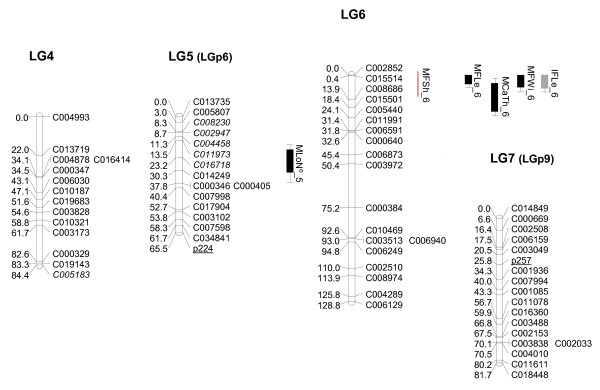
**Genetic map of Zucchini × Scallop F_2 _population (LG4, LG5, LG6, LG7)**. Linkage map and locations of quantitative trait *loci *(QTL) whose effects have been verified in the backcross populations associated with vine development, flowering and fruit quality based on 146 F_2 _plants derived from a Zucchini × Scallop cross. The linkage groups (LGs) have been ordered according to the results obtained in this paper. Group numbers in parenthesis (LGp) correspond to LGs in the map by Gong et al. [44]. The correspondence between the two linkage groups has been determined according to the common SSR markers between maps (underlined). Markers with distorted segregation in F_2 _are in italics. QTL indicated in light grey, grey or black correspond to flowering, immature of mature fruit traits, respectively. QTL are represented with bars (2-LOD interval) and boxes (1-LOD interval). QTL for qualitative traits are represented with red lines spanning the region between flanking markers significantly associated with the trait at P < 0.05.

**Figure 4:**
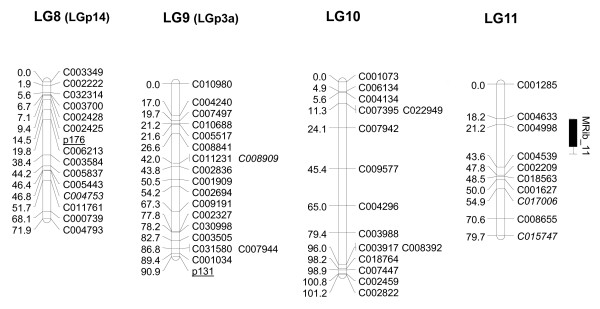
**Genetic map of Zucchini × Scallop F_2 _population (LG8, LG9, LG10, LG11)**. Linkage map and locations of quantitative trait *loci *(QTL) whose effects have been verified in the backcross populations associated with vine development, flowering and fruit quality based on 146 F_2 _plants derived from a Zucchini × Scallop cross. The linkage groups (LGs) have been ordered according to the results obtained in this paper. Group numbers in parenthesis (LGp) correspond to LGs in the map by Gong et al. [44]. The correspondence between the two linkage groups has been determined according to the common SSR markers between maps (underlined). Markers with distorted segregation in F_2 _are in italics. QTL indicated in light grey, grey or black correspond to flowering, immature of mature fruit traits, respectively. QTL are represented with bars (2-LOD interval) and boxes (1-LOD interval). QTL for qualitative traits are represented with red lines spanning the region between flanking markers significantly associated with the trait at P < 0.05.

**Figure 5:**
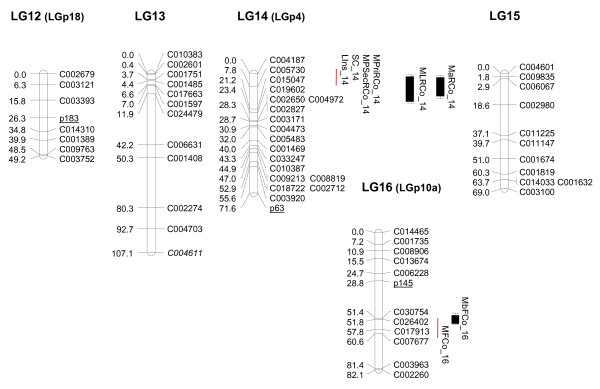
**Genetic map of Zucchini × Scallop F_2 _population (LG12, LG13, LG14, LG15, LG16)**. Linkage map and locations of quantitative trait *loci *(QTL) whose effects have been verified in the backcross populations associated with vine development, flowering and fruit quality based on 146 F_2 _plants derived from a Zucchini × Scallop cross. The linkage groups (LGs) have been ordered according to the results obtained in this paper. Group numbers in parenthesis (LGp) correspond to LGs in the map by Gong et al. [44]. The correspondence between the two linkage groups has been determined according to the common SSR markers between maps (underlined). Markers with distorted segregation in F_2 _are in italics. QTL indicated in light grey, grey or black correspond to flowering, immature of mature fruit traits, respectively. QTL are represented with bars (2-LOD interval) and boxes (1-LOD interval). QTL for qualitative traits are represented with red lines spanning the region between flanking markers significantly associated with the trait at P < 0.05.

**Figure 6:**
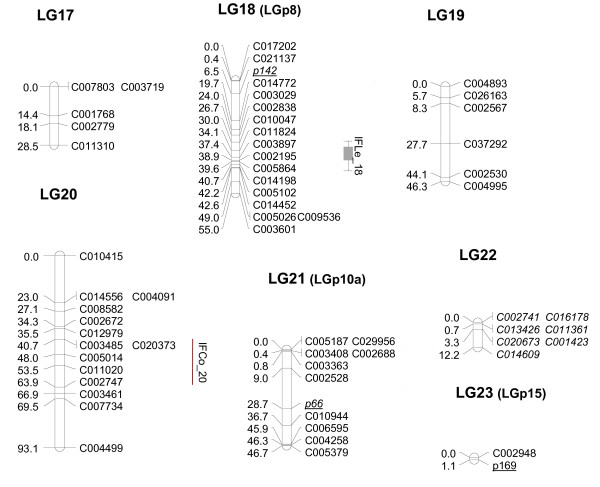
**Genetic map of Zucchini × Scallop F_2 _population (LG17, LG18, LG19, LG20, LG21, LG22, LG23)**. Linkage map and locations of quantitative trait *loci *(QTL) whose effects have been verified in the backcross populations associated with vine development, flowering and fruit quality based on 146 F_2 _plants derived from a Zucchini × Scallop cross. The linkage groups (LGs) have been ordered according to the results obtained in this paper. Group numbers in parenthesis (LGp) correspond to LGs in the map by Gong et al. [44]. The correspondence between the two linkage groups has been determined according to the common SSR markers between maps (underlined). Markers with distorted segregation in F_2 _are in italics. QTL indicated in light grey, grey or black correspond to flowering, immature of mature fruit traits, respectively. QTL are represented with bars (2-LOD interval) and boxes (1-LOD interval). QTL for qualitative traits are represented with red lines spanning the region between flanking markers significantly associated with the trait at P < 0.05.

We were able to successfully map 304 of the 330 SNP that were polymorphic between the parentals. A set of 26 validated markers was discarded for mapping, either because the SNP did not show the three genotypic classes in the F_2 _or because one of the parents was heterozygous. The genetic map was constructed using a set of 315 high-quality markers (304 SNP and 11 SSR) using MAPMAKER at a LOD score of 4 (Figures 2, 3, 4, 5 and 6). The MU-CU-16 × UPV-196 genetic map covered 1,740.8 cM and was divided into 22 major linkage groups (LGs) and a minor group (LG23, with only 2 markers, 1.1 cM), with an average of 6.02 ± 6.65 cM between markers. The maximum gap between markers was 30.3 cM in LG13. Two SNP, C007167 and C008395, remained unlinked.

The total number of markers included in major LGs varied from 5 in LG17 to 31 in LG2. Apart from LG23, only three groups contained less than eight markers (LG17, LG19 and LG22), with the markers being more or less evenly distributed among and within each LG group. LG length ranged from 12.2 cM in LG22 to 173.8 cM in LG2. On average, a linkage group covered 79.1 ± 34.7 cM and contained 14.1 ± 5.8 markers, resulting in an average map density of 5.56 ± 1.70 cM/marker. Less coverage was presented herein in comparison to the previous map for the species (1,936 cM and a density of 2.9 cM/marker) [44]. However, the previous map was mainly constructed with dominant, non-transferable RAPD or AFLP. Of the 659 *loci *mapped, only 178 correspond to co-dominant SSR, which appeared unevenly distributed across the genome. Our results with the transferability of these markers have also been very low. The SNP-based map presented here is the first to include high-quality markers amenable to automation in the genus *Cucurbita*, many of which are putatively transferable to other populations and even to other species, and most of which are in fully annotated genes involved in diverse biological processes. In addition, distances have been reported not to be comparable between different software, as Joinmap lengths of the individual linkage groups are usually shorter than those obtained with MAPMAKER [66,67,44].

Distorted segregation was observed in 30 SNP and 2 SSR, a larger number than in the Pumpkin × Crookneck cross [43,44], but lower than that reported in maps constructed from interspecific crosses [41]. Grouped markers were especially observed in LG2 and LG5 (Figures 2 and 3).

Using the microsatellites as anchors to the previous *Cucurbita *map, it was possible to associate the linkage groups of both maps: LG2, LG5, LG7, LG8, LG9, LG12, LG14, LG16, LG18, LG21 and LG23 correspond to groups LGp2, 6, 9, 14, 3a, 18, 4, 10a, 8, 10a and 15 from Gong et al. [44], respectively. In the previous map, CMTp145 and CMTp66 mapped in the same group (10a) at LOD 3, but in this study, they appear associated with different groups (LG16 and LG21). In the future, newly developed SNP will have to be mapped to improve the map saturation and obtain the 20 expected linkage groups, merging some of those that are less represented in the current map.

The distorted segregation found in LG2 was not reported in the corresponding LGp2 [44], even though only three markers were mapped in this linkage group and the anchor SSR mapped in LG2 is out of this area. Scallop alleles were over-represented, suggesting that the alleles in this region may be subject to gametic or zygotic selection and/or related to preferential germination or better seedling viability. Different functions were associated with the distorted markers (Additional File 3). Some of these unigenes may be the cause of the segregation distortion, but it could also be the result of linkage to other genes.

### Synteny with cucumber

**Figure 7:**
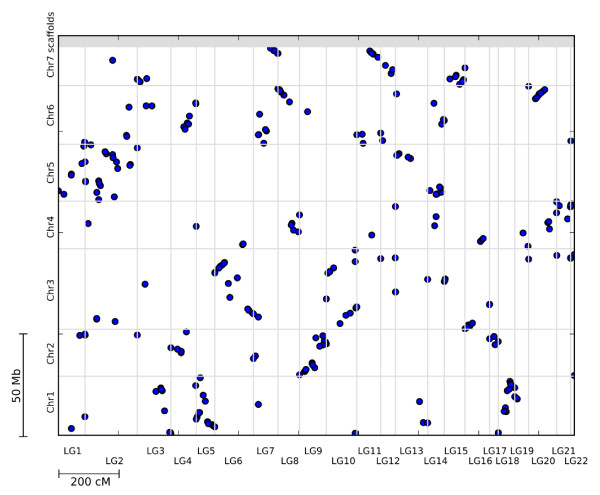
**Synteny with cucumber**. Graphical representation of the tBLASTx hits found between the cucumber (Y-axis) chromosomes and the *Cucurbita *linkage groups (X-axis). Both the chromosomes and the linkage groups are numerically sorted in their respective axis and their limits are shown by grey lines. Only the tBLASTx hits with an e-value above 10-^6 ^are shown in the figure.

Three hundred of the 304 mapped unigenes, yielded significant tBLASTx hits (threshold e-value of 10^-6^) and were assigned to the cucumber chromosomes. Figure 7 shows the colinearity between the genomes of the two species, *C. sativus *and *C. pepo*; details about the position of the unigenes in the cucumber genome are also included in Additional File 3. We found syntenic blocks between most of the *C. pepo *linkage groups and *C. sativus *chromosomes.

Syntenic studies in the family Cucurbitaceae have been conducted with the two main cultivated species of the *Cucumis *genus: cucumber (2n = 14) and melon (2n = 24). Recent studies, using common markers and the whole genome sequence of cucumber, have shown that colinearity exists between cucumber and melon, indicating that chromosome fusions and other complex structural changes have generated cucumber chromosomes from a progenitor species with 2n = 24 [68]. We also found a high level of colinearity between *C. pepo *and the cucumber genome. Some *Cucurbita *linkage groups (LG) can be considered homoeologous to cucumber chromosomes (Chr). For example, *Cucurbita *LGs 3, 5 and 18 showed syntenic blocks with cucumber Chromosome 1, LG9 and 17 with Chr2, LG6 and 10 with Chr3, LG21 with Chr4, LG1, 2 and 14 with Chr5, LG7 with Chr6, and LG11 and 15 with Chr7. Some of the remaining LGs (4, 8, and 20) were syntenic to genetic blocks from two cucumber chromosomes (Chr 2-6, and Chr 4-6).

Most cucumber chromosomes contained two to three partially overlapping syntenic blocks with different LGs of *C. pepo*, which may suggest a certain level of duplication in this species. The higher chromosome number (2n = 40) of *Cucurbita *suggests that this genus may be of polyploid origin. In fact, previous cytogenetic and isozyme studies indicate that this genus may be an ancient tetraploid [69,70]. Our results agree with a certain degree of duplication in this species.

A recent study on the level of macrosynteny between two species of the genus, *C. pepo *and *C. moschata*, through a comparative alignment of SSR markers, did not provide any indication of a possible ancient polyploid origin of the species [45]. In that paper, the authors studied the segregation of SSR *loci*, previously selected to be uniquely located in the genome. However, in our study, synteny has been analyzed by blasting whole unigene sequences, which is more likely to yield significant matches in diverse genome sites than the uniquely located SSR primers. Differences in the approaches and the higher number of markers used in our study may explain the differences between the BLAST-based and SSR-based results.

### QTL identification and QTL effect validation for Marker-Assisted Selection

Additional File 5: "Quantitative and qualitative traits" shows the values found for each attribute in the parents, F_2 _and the backcross populations, clearly demonstrating phenotypic variability for most attributes. Forty-eight QTL were detected for 31 quantitative traits and 11 QTL were detected for 11 qualitative traits. These QTL were distributed in 24 independent positions in 13 linkage groups. The proportion of the phenotypic variance explained by a single QTL (R^2^) varied from 7% to 81%. Fifteen major QTL (R^2^> 25%) were detected for flowering traits (associated with late flowering and maleness tendency) and for immature and mature fruit traits (associated with fruit length and rind and flesh color). Detailed information about all these QTL (explained variance, LOD peaks, flanking markers, additive and dominance effects and heritabilities) are shown in Additional Files 6 and 7: "QTL analysis for quantitative and qualitative traits 1 and 2".

The genetic inheritance of important agronomic traits is largely unknown in *Cucurbita*. This QTL analysis provides the first results of the genetic control of most of these plant, flowering and fruit traits. Our preliminary results should be further confirmed using additional populations and phenotypic replications. In this paper, we confirmed the utility of some of these QTL for Marker-Assisted Selection by validating their effects on the backcross populations. Despite the limited number of plants, the effects of eleven of the 15 major QTL (*NoMaF_3, NoFeF_3, DMaF_3, DFeF_3, N°MaF_3, MaF/FeF_3, IFLe_6, MFLe_6, MLRCo_14, MaRCo_14 *and *MbFCo_16*) detected in the F_2 _were verified in one or both backcross populations (Table 2). In addition, six minor QTL (all with R^2^> 10%) (*IFLe_18, MFWi_6, MRib_11, MCaTh_6, MFFi_2 *and *MLoN°_5) *and eight QTL involved in qualitative traits (*SC_14, LIns_14, T_1, IFCo_20, MFSh_6, MPriRCo_14, MPSecRCo_14 *and *MFCo_16*) were also verified in the backcrosses (Table 2). The verified QTL segregated differently between the backcross populations, segregating only in one or in both of them. This differential segregation is in general compatible with the direction of additive effects and dominance deviation estimated in the F_2_. Information about the QTL set validated in the backcross populations is detailed in Additional File 7. The most likely positions on the linkage map for these validated QTL are shown in Figures 2, 3, 4, 5 and 6. The most important QTL displaying real effects in backcrosses related to flowering, fruit shape and color are described below in greater detail.

**Table 2:** QTL effects validated in backcross populations

A Quantitative traits					
**QTL**	**Marker^1^**	**BCZ^2^**		**BCS^3^**	
		
		**a**	**h**	**b**	**h**

*Flowering*					

***NoMaF_3***	C001057	1.273	1.143	**3.467**	**1.929**

***NoFeF_3***	C001057	11.546	9.714	**19.400**	**14.286**

***DMaF_3***	C001057	24.818	23.571	**30.200**	**26.786**

***DFeF_3***	C001057	31.364	27.571	**41.000**	**36.357**

***N°MaF_3***	C001057	19.000	21.000	**35.300**	**30.800**

***MaF/FeF_3***	C001057	3.974	2.531	**18.900**	**11.600**

*Immature fruits*					

***IFLe_6***	C002852	**19.633**	**15.450**	**7.890**	**11.556**

*IFLe_18*	C003897	17.717	17.367	**8.300**	**10.500**

*Mature fruits*					

***MFLe_6***	C002852	**31.806**	**26.994**	**9.728**	**13.386**

*MFWi_6*	C002852	**9.361**	**10.950**	13.469	13.250

*MRib_11*	C004998	0.000	0.000	**2.118**	**1.500**

*MCaTh_6*	C008686	**61.847**	**75.932**	80.080	85.575

*MFFi_2*	C011474	10.614	10.830	**11.648**	**8.671**

*MLoN°_5*	C016718	3.450	3.500	**4.600**	**3.900**

***MLRCo_14***	C005730	**50.285**	**77.989**	**82.382**	**78.662**

***MaRCo_14***	C005730	**-7.902**	**0.419**	-0.4406	0.9755

***MbFCo_16***	C030754	22.338	26.532	**10.636**	**12.139**

**B **Qualitative traits^4^					

QTL	Marker^1^	BCZ^2^		BCS^3^	
	
		a	H	b	h

*Vine traits*					

*SC_14*	C005730				

dark green		0.64	0.29	**0.18**	**0.67**

intermediate		0.36	0.71	**0.64**	**0.33**

light green		0.00	0.00	**0.18**	**0.00**

P^5^		0.192		**0.011†**	

*LIns_14*	C005730				

absent		**1.00**	**0.29**	0.00	0.00

present		**0.00**	**0.71**	1.00	1.00

P		**0.020†**		1.00	

*T_1*	C003546				

present		0.58	0.33	**0.53**	**1.00**

absent		0.42	0.67	**0.47**	**0.00**

P		0.864		**0.011†**	

*Immature fruits*					

*IFCo_20*	C005014				

green		**0.13**	**0.00**	0.00	0.00

light green		**0.00**	**0.50**	0.00	0.08

white-green		**0.88**	**0.50**	0.17	0.46

white		**0.00**	**0.00**	0.83	0.46

P		**0.051†**		0.404	

*Mature fruits*					

*MFSh_6*	C002852				

elongated		1.00	0.89	**0.00**	**0.00**

pear-shaped		0.00	0.11	**0.06**	**0.36**

discoid		0.00	0.00	**0.94**	**0.64**

P		0.500		**0.042†**	

*MPriRCo_14*	C005730				

black		**0.91**	**0.00**	0.00	0.00

green		**0.09**	**0.00**	0.00	0.00

cream		**0.00**	**0.29**	0.00	0.06

white		**0.00**	**0.71**	1.00	0.94

P		**0.000†**		1.000	

*MPSecRCo_14*	C004187				

dotted speckled		**0.92**	**0.50**	0.17	0.00

banded		**0.00**	**0.00**	0.00	0.00

striped		**0.00**	**0.00**	0.00	0.00

absent		**0.08**	**0.50**	0.83	1.00

P		**0.022†**		0.163	

*MFCo_16*	C017913				

green		**0.09**	**0.00**	0.00	0.00

orange		**0.45**	**0.00**	0.00	0.00

yellow		**0.27**	**0.67**	0.00	0.00

cream		**0.09**	**0.00**	0.15	0.00

white		**0.09**	**0.33**	0.85	1.00

P		**0.051†**		0.222	

**A**. Average data for quantitative traits with QTL displaying significant differences (P > 0.05) between homozygous and heterozygous individuals for linked markers in BC populations. Major QTL (R^2^> 25%) are indicated in bold. Data traits with significant differences are indicated in bold. **B**. Frequency for the different categories for qualitative traits with QTL displaying significant differences between homozygous and heterozygous individuals for linked markers in BC populations and results of the Fisher exact test. Data traits with significant differences are indicated in bold.

**^1 ^**Tested markers located in the QTL region (see Figures 2, 3, 4, 5 and 6).

^2 ^Homozygotes for the Zucchini allele of the corresponding marker in BCZ are indicated as a (allele from MU-CU-16), while heterozygotes are indicated as h.

^3 ^Homozygotes for the Scallop allele of the corresponding marker in BCS are indicated as b (allele from UPV-196), while heterozygotes are indicated as h.

^4 ^Only the categories represented in the BC populations are included.

^5 ^Fisher's exact probability test. P (α = 0.05). P < 0.05 implies association and linkage with the marker, as H_0 _of independence is rejected (†).

#### Flowering

A cluster of QTL controlling several flowering traits (all with medium-high broad-sense heritabilities,0.71 - 0.85) was detected in LG3, most of which had major effects (R^2^> 25%) and partial or complete dominance of the Zucchini alleles (d/[a] from -0.78 to -1.05), associated with the early appearance of male and female flowers as well as an enhanced femaleness tendency of the plant (*NoMaF_3, DMaF_3, NoFeF_3, DFeF_3, N°MaF_3, MaF/FeF_3*) (Additional File 7, Figure 2). In agreement with the a and d values estimated in the F_2_, the backcrosses show how the Scallop alleles delayed flowering and increased maleness with a recessive gene action (Table 2). Consequently, no differences between plants homozygous for the Zucchini alleles *versus *heterozygous were found in the BCZ population, whereas the mean of the plants homozygous for the Scallop alleles was significantly higher than those of the heterozygous genotypes in the BCS population. The sex expression in Cucurbitaceae is known to be controlled by various genetic, environmental and hormonal factors, with ethylene being the main hormone involved in this trait. In *C. sativus *and *C. melo*, it is controlled by several major independent genes, some of which have been cloned [71-73]. Our results also suggest the existence of a major gene controlling flowering time and the enhanced female/maleness phenotype in summer squash. Further research is necessary to determine whether the co-segregation of the flowering time traits and female/male tendency is due to pleiotropy at a single *locus *or linkage between *loci*.

#### Fruit shape

Two major QTL (R^2^> 25%) involved in fruit shape, controlling the length of immature and mature fruits (*IFLe_6 *and *MFLe_6*), co-segregate in LG6, along with various minor QTL that control mature-fruit width and cavity thickness (*MFWi_6, MCaTh_6*) and also with a QTL controlling fruit shape (MFSh_6) (Additional File 7, Figure 3). The Zucchini type contributed alleles producing elongated fruits, while the Scallop alleles produced wider fruits with wider cavities. Most of these traits presented moderate heritabilities. The two major QTL (*IFLe_6 *and *MFLe_6*), with additive gene action estimated in the F_2 _(d/[a] 0.24 and -0.09 respectively), were verified in both the BCS and BCZ populations with the expected direction of allelic effects (Table 2). These results suggest that these QTL can be exploited in both genetic backgrounds for hybrid or pure line development. *MFWi_6 *and *MCaTh_6 *were also additive in the F_2_, but they were verified only in one of the backcross populations, which may be due to the low capacity for QTL detection in the backcross populations due to their modest sample size or to genetic background effects. An independent QTL for fruit length was detected in LG18 (*IFLe_18*). Also, Scallop alleles of *MLoN°_5 *and *MRib_11 *modified fruit shape by increasing the number of locules and the ribbing intensity.

Several genes have been reported to be related to fruit shape. A dominant gene (*Di*) seems to control the discoid fruit shape of scallop squash [46]. This gene was reported to be dominant over spherical or pyriform shapes. A digenic epistatic control has also been reported for summer squash fruit shape. Our results are consistent with the existence of a major gene that is, however, not dominant, and several minor modifiers.

#### Fruit color

Major QTL for the rind color of mature fruits mapped in LG14 (*MLRCo_14 *and *MaRCo_14*), with lightness (L Hunter parameter, white color) increasing with Scallop alleles and greenness increasing with Zucchini alleles (Additional File 7, Figure 5). High heritabilities were found (0.95 and 0.97) for these rind color parameters. Also, the visual scores of primary rind color and the pattern of secondary color mapped in the same region (*MPriRCo_14 *and *MPSecRCo_14)*, with Zucchini alleles leading to dark rind colors and Scallop alleles to the absence of a secondary color (Table 2).

The genetic control of flesh color seems to be independent. A major QTL for immature flesh color is located in LG20 (*IFCo_20*), whereas a major QTL was found for mature fruit flesh color in LG16 (*MbFCo_16*), which is consistent with the location of the qualitative trait color *MFCo_16 *(Additional File 7, Figures 5 and 6).

Squash fruit color has been studied intensively, and a complex genetic control has been proposed for rind color, with major genes (one dominant, derived from Scallop *W *(weak rind coloration)) [46], complemented by modifiers, whereas less complexity is reported for flesh color. The QTL that control rind color in mature fruits were validated in one or both backcrosses (Table 2). Plants homozygous for the Zucchini allele are dark green or black, whereas individuals that are heterozygous or homozygous for the Scallop allele are white or cream-colored in any genetic background, consistently with the major gene *W*, which confers a white or cream color independently of genetic background [46]. This gene has been reported to be complementary to the major gene, *Wf*, also from Scallop, which is dominant over colored flesh [46], as most white-rinded squashes are also white-fleshed. Accordingly, mature homozygous Zucchinis for the SNP marker C017913, which is linked to *MFCo-16*, were mostly orange/yellow-fleshed, whereas homozygous Scallops were mostly white-fleshed. However, heterozygous individuals were all white-fleshed in the Scallop background, while some yellow-fleshed fruits appeared in the Zucchini background. Consistently, a significant effect of Scallop alleles of C030754 (linked to *MbFCo_16*), reducing flesh yellowness, was only detected in the Scallop background. Therefore, it seems to be a major gene with dominance of white flesh (*Wf*), although other minor genes also seem to contribute to the control of flesh color.

Most of the QTL reported in this paper had not been located previously. The maps that have been developed to date include six monogenic traits (precocious yellow fruit, *B*; bush growth habit, *Bu; *leaf mottling, *M*; hull-less seed coat, *n; *and mature fruit color) [41,42,44], most of which did not segregate in our population. QTL for fruit length, width and number of fruit locules were located on a Zucchini × Crookneck map constructed using RAPD markers [42]. Other QTL were also reportedly associated with RAPD markers for fruit shape and leaf indentation using an interspecific *C. pepo *x *C*. *moschata *map [41]. However, the comparison of the results is not possible due to the lack of common markers with the current map. These previously detected QTL have not been used to date for MAS selection in *Cucurbita*.

## Conclusions

Our results demonstrate the utility of the 384-SNP GoldenGate genotyping array in *Cucurbita pepo*. Next-generation sequencing, together with this cost-effective genotyping technique, have been successfully applied to constructing the first SNP-based genetic map reported in the genus. This Zucchini × Scallop map is not only an important resource for high-quality markers that are polymorphic between two highly contrasting squash types, but is also an invaluable tool for breeding purposes, since these markers are developed in coding regions involved in different physiological processes. Several preliminary QTL related to vine, flowering and fruit traits in the mature and immature stages have been reported and mapped for the first time. QTL effects have been validated as has been the utility of various markers for marker-assisted selection, which demonstrates the suitability of the current population and genetic map for dissecting genetically complex fruit traits in *Cucurbita *ssp. This information will be essential for future breeding programs focused on obtaining better-adapted varieties. The SNP platform has been successfully assayed to detect variability between/within both *C. pepo *subspecies and different squash morphotypes, and has also revealed a great number of *loci *transferable to *C. moschata*. This will facilitate synteny studies with other cucurbits and subsequent diversity and mapping studies that will contribute to increasing the genomic resources for these crops.

## Authors' contributions

BP designed and coordinated the study, developed the F_2 _population, selected the markers for the GoldenGate assay, provided the phenotypic data and contributed to the mapping, QTL and synteny analysis. CE participated in the design of the study, and in the development of the F_2 _population, and contributed to the phenotyping process. CE prepared the DNA of the populations, contributed to the SNP selection process, and performed the SSR analysis. AJM and CE performed the mapping and QTL analysis. JB assisted in the marker selection and in the synteny study. CR assisted in the annotation process. PG participated in the conception of the study. PG and NVD also participated in the design and development of the F_2 _population, and contributed to the phenotyping and genotyping processes. FN is the director of the COMAV and participated in the conception of the study. BP and CE integrated all the information and drafted the manuscript, with contributions from all authors. All authors have read and approved the final manuscript.

## Supplementary Material

Additional file 1 **Sequence and primers for genotyping the 384 SNP included in the GoldenGate platform**. The sequences of each of the 384 SNP included in the GoldenGate platform are indicated, including the polymorphic nucleotide and a 60 bp flanking sequence, along with the *locus*-specific and two allele-specific primers designed for detecting each *locus *with the GoldenGate assay, the three universal primers and the Illumicode. Click here for file

Additional file 2 **Primers for genotyping the SSR included in the map**. The primers for genotyping the 25 SSR selected from the previous *C. pepo *map [44] are listed, along with the genotyping results and map position for each *locus*. Click here for file

Additional file 3 **Annotation data and map position of the 384 *loci *included in the GoldenGate platform**. Annotation data of the 384 unigenes included in the GoldenGate platform are described. Previous annotation data provided by Blanca et al. [20], after *in silico *detection of the unigenes, consist of unigene length, position of the SNP, in ORF or UTR, number of reads in each parental, GO terms, gene description after sequential BLAST of Swissprot, Arabidopsis org and Uniref90 [51-53], orthologs detected with *Arabidopsis *and *C. melo *by reciprocal BLAST of *Arabidopsis*_pep and ICUGI databases [52,17] and putative SNP-CAPS. Data generated in this paper are final CRG/CEGEN scores for GoldenGate genotyping reactions, GoldenGate genotyping results, distribution of the unigenes in the *C. sativus *genome (scaffolds or chromosomes) after BLAST against cucumber genome available at ICUGI [17], linkage group according to the Zucchini × Scallop map obtained, variability of SNP in the germplasm panel of *C. pepo *subsp. *pepo *and subsp. *ovifera *accessions and amplification in *C. moschata*. Click here for file

Additional file 4 **Number of unigenes in each functional category**. Number of unigenes, of the 384 included in the GoldenGate platform, assigned to each GO Slim in the Biological Process category (A) and the Molecular Function category (B). Click here for file

Additional file 5 **Quantitative and qualitative traits**. **A**. Scored quantitative traits are described. Mean values, ranges and standard deviation for parental, F_2 _and backcross populations are indicated. **B**. Pearson correlations between pairs of quantitative traits. **C**. Visually scored qualitative traits are described. Relative frequency of each phenotype is shown for both parental, F_2 _and backcross population. Click here for file

Additional file 6 **QTL analysis for quantitative and qualitative traits 1**. QTL whose effects have not been validated in the backcross populations are included. **A**. Linkage group positions and flanking markers of 31 QTL, along with their associated logarithms of odds (LOD) for 20 vine, flowering and fruit quantitative traits analyzed in the F_2 _population derived from the Zucchini × Scallop cross. Major QTL (R^2^> 25%) are indicated in bold. **B**. Linkage group positions and flanking markers of 3 QTL, along with their associated logarithms of odds (LOD) and contingency χ^2 ^results for fruit qualitative traits analyzed in the F_2 _population derived from the cross Zucchini × Scallop. Major QTL (R^2^> 25%) are indicated in bold. Click here for file

Additional file 7 **QTL analysis for quantitative and qualitative traits 2**. QTL whose effects have been validated in the backcross populations are included. **A**. Linkage group positions and flanking markers of 17 QTL, along with their associated logarithms of odds (LOD) for 16 flowering and fruit quantitative traits analyzed in the F_2 _population derived from the Zucchini × Scallop cross. Major QTL (R^2^> 25%) are indicated in bold. Heritabilities have been calculatedfor these traits **B**. Linkage group positions and flanking markers of 8 QTL, along with their associated logarithms of odds (LOD) and contingency χ^2 ^results for vine and fruit qualitative traits analyzed in the F_2 _population derived from the cross Zucchini × Scallop. Major QTL (R^2^> 25%) are indicated in bold. Click here for file
