# Supplementary figures and images for: High-throughput SNP genotyping in Cucurbita pepo for map construction and quantitative trait loci mapping
Source: BMC Genomics. 2012 Feb 22;13:80. doi: 10.1186/1471-2164-13-80 (PMC3359225; doi:10.1186/1471-2164-13-80)

## Slide 1
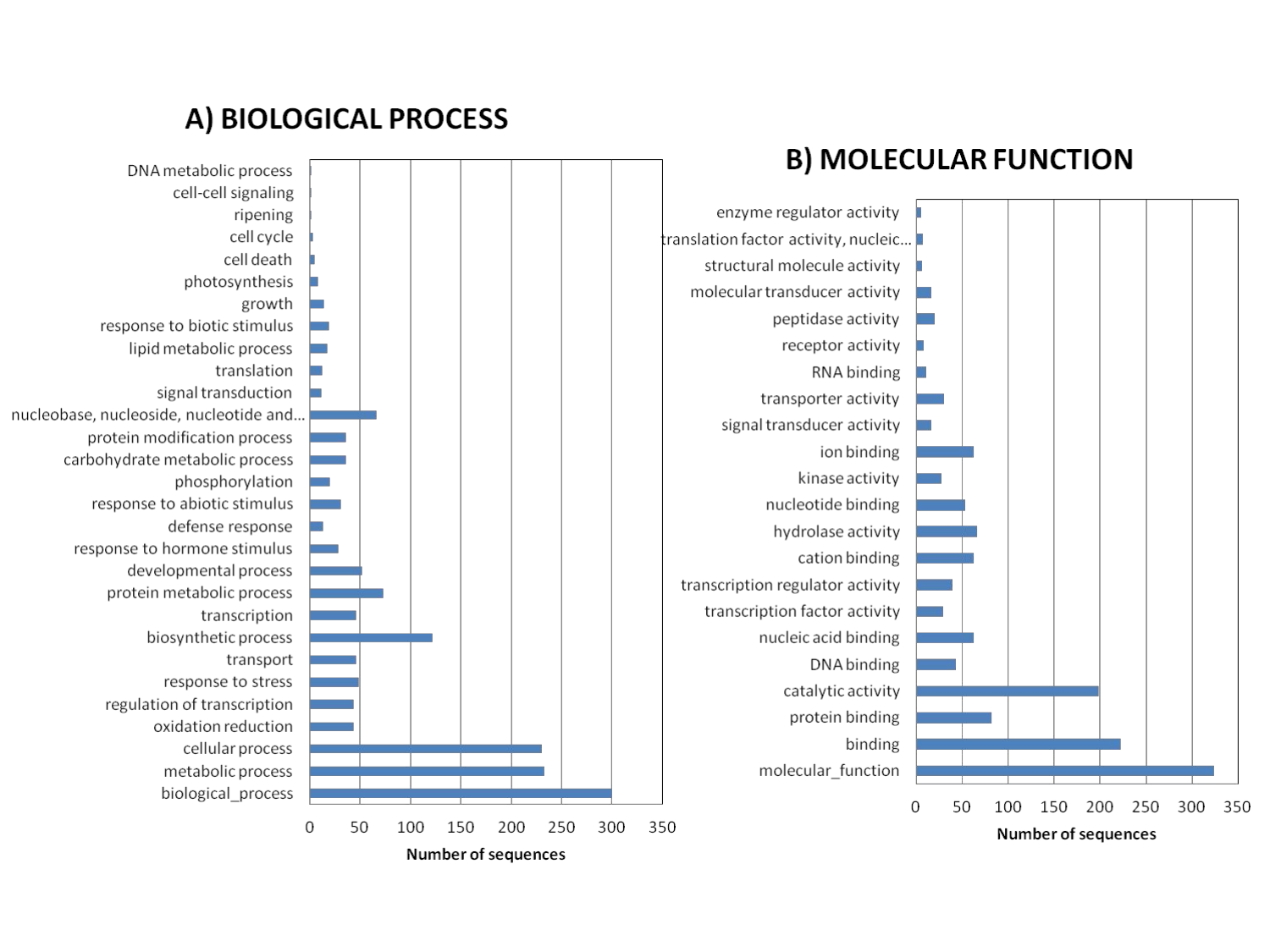

Supplement: Additional file 4 — Number of unigenes in each functional category. Number of unigenes, of the 384 included in the GoldenGate platform, assigned to each GO Slim in the Biological Process category (A) and the Molecular Function category (B). [file 1471-2164-13-80-S4.PPT]
